# Genomic variation associated with local adaptation of weedy rice during de-domestication

**DOI:** 10.1038/ncomms15323

**Published:** 2017-05-24

**Authors:** Jie Qiu, Yongjun Zhou, Lingfeng Mao, Chuyu Ye, Weidi Wang, Jianping Zhang, Yongyi Yu, Fei Fu, Yunfei Wang, Feijian Qian, Ting Qi, Sanling Wu, Most Humaira Sultana, Ya-Nan Cao, Yu Wang, Michael P. Timko, Song Ge, Longjiang Fan, Yongliang Lu

**Affiliations:** 1 Institutue of Crop Science & Institute of Bioinformatics, College of Agriculture and Biotechnology, Zhejiang University, Hangzhou 310058, China; 2 China National Rice Research Institute, Chinese Academy of Agricultural Sciences, Hangzhou 310006, China; 3 Zhejiang Sheng Ting Biotechnology Co., Ltd., Taizhou 318020, China; 4 Analysis Center of Agrobiology and Environmental Sciences, Faculty of Agriculture, Life and Environmental Sciences, Zhejiang University, Hangzhou 310058, China; 5 Key Laboratory of Conservation Biology for Endangered Wildlife of the Ministry of Education, Zhejiang University, Hangzhou 310058, China; 6 Department of Biology, University of Virginia, Charlottesville, Virginia 22904, USA; 7 State Key Laboratory of Systematic and Evolutionary Botany, Institute of Botany, Chinese Academy of Sciences, Beijing 100093, China

## Abstract

De-domestication is a unique evolutionary process by which domesticated crops are converted into ‘wild predecessor like' forms. Weedy rice (*Oryza sativa f. spontanea*) is an excellent model to dissect the molecular processes underlying de-domestication. Here, we analyse the genomes of 155 weedy and 76 locally cultivated rice accessions from four representative regions in China that were sequenced to an average 18.2 × coverage. Phylogenetic and demographic analyses indicate that Chinese weedy rice was de-domesticated independently from cultivated rice and experienced a strong genetic bottleneck. Although evolving from multiple origins, critical genes underlying convergent evolution of different weedy types can be found. Allele frequency analyses suggest that standing variations and new mutations contribute differently to *japonica* and *indica* weedy rice. We identify a Mb-scale genomic region present in weedy rice but not cultivated rice genomes that shows evidence of balancing selection, thereby suggesting that there might be more complexity inherent to the process of de-domestication.

## Abstract (web summary)

De-domestication is the process by which cultivated plants adopt characteristics similar to that of their wild predecessors. Here Qiu *et al*. re-sequence de-domesticated weedy rice and matched cultivated varieties and identify genetic variants indicative of convergent evolution across multiple de-domestication events.

Plant domestication is the process during which wild species are converted into crop plants through artificial selection. Both conscious and unconscious selections by humans during this process have brought about significant alterations of plant traits to meet their desires and benefits^1^. Domestication can result in increased fitness of a plant under human cultivation, and often decreases its viability in the natural environment as well^2^. Plant de-domestication is a distinct evolutionary process involving a loss of traits aggregated under domestication, during which domesticated crops are turned into self-sustainable ‘wild-like' plants mainly driven by natural selection^3^,^4^,^5^. Ellstrand *et al*.^3^ conducted a survey in search of examples of plants that originated by de-domestication. Based on strict criteria only 13 examples of de-domestication could be found, all of which shared the common feature that their descendants were weedy or invasive plants with intrinsic capacity for rapid adaptation to their living environments. Compared with the well-studied process of domestication, the mechanism of de-domestication has not been thoroughly investigated. A recent study on feral chicken populations indicated that de-domestication targets different genomic loci to domestication^6^. The universality of the population genetic process during de-domestication needs to be examined. The descendants of de-domestication originated from crops with relatively low genetic diversity^7^. When returning to the feral environment, whether they just employ limited standing variations or generate new mutations for their survivals still remains unknown. In addition, human-imposed directional selection drives the rapid evolution of domesticates during domestication. However, when domesticates are back to feral environments with more diverse pressures, what other selection regimes may exist? Therefore, a significant opportunity exists to examine how evolutionary processes act on the genomic and molecular level for the rapid adaptation of the decedents of domesticated species during de-domestication.

Weedy rice (*Oryza sativa f. spontanea*), also called ‘red rice', is a conspecific weed of cultivated rice. Although multiple origins for weedy rice have been proposed, currently de-domestication from cultivated varieties has been acknowledged as one of the main routes for the origin of weedy rice all over the world^8^,^9^,^10^,^11^,^12^,^13^,^14^. This kind of weed is also one of the plants identified by Ellstrand *et al*.^3^ as having originated by de-domestication. Compared with the other species, domesticated rice genome assembly and its annotation provide good proxies and facilitate the work on weedy rice^15^,^16^,^17^. Therefore, weedy rice may serve as one of the best models to investigate the process of crop de-domestication.

It is well established that after following its evolution from domesticated rice, weedy rice becomes an invasive plant and pernicious pest in paddy fields^18^,^19^,^20^. Weedy rice harbours many growth characters more similar to wild rice forms than domesticates that enhance its survival abilities. For example, weedy rice has a shattering phenotype that readily releases seeds to the soil where they can persist dormancy for many years^18^. On the other hand, given its close genetic relationship with cultivated rice, the development of herbicides that can control weedy rice without injuring commercial rice has proven difficult, resulting in weedy rice being as a continued serious constraint to rice production worldwide^20^.

Up to now, our understanding of the underlying adaptive mechanisms of weedy rice during de-domestication remains quite limited. Several previous studies have provided helpful insights into this process^9^,^10^,^11^,^12^,^13^,^14^,^19^,^21^. For instance, Reagon *et al*.^19^ examined the *SD1* gene and were able to demonstrate a critical role of introgressive hybridisation during the divergence of descendant weedy rice from its cultivated progenitor. Qi *et al*.^21^ used genotyping by sequencing to identify multiple small-to-moderate effect quantitative trait loci for weediness traits important in weedy rice forms compared with cultivated forms and suggested that weedy rice may be a product of parallel evolution through independent genetic mechanisms. However, a broad understanding of the genomic scale changes occurring during de-domestication has yet to be developed.

To address this paucity of information, in this present study we sequenced the genomes of individual weedy rice sample from four representative populations across China and the contiguously growing cultivated rice forms in these regions. Our results indicated that the Chinese weedy rice was de-domesticated independently from cultivated rice and suffered a genetic bottleneck during each de-domestication event based on phylogenetic and demographic analyses. Genomic signatures of weedy rice identified in our work allowed us to (i) define the critical genes that underlie convergent evolution in different populations given the fact that different weedy groups evolve independently in local environments; (ii) determine the relative roles of standing variations and new mutations in the rapid adaptation of weedy rice during de-domestication; and (iii) determine what kind of selection is occurring during the de-domestication process.

## Results

### Phenotypes and genome re-sequencing of weedy rice

**Figure 1:**
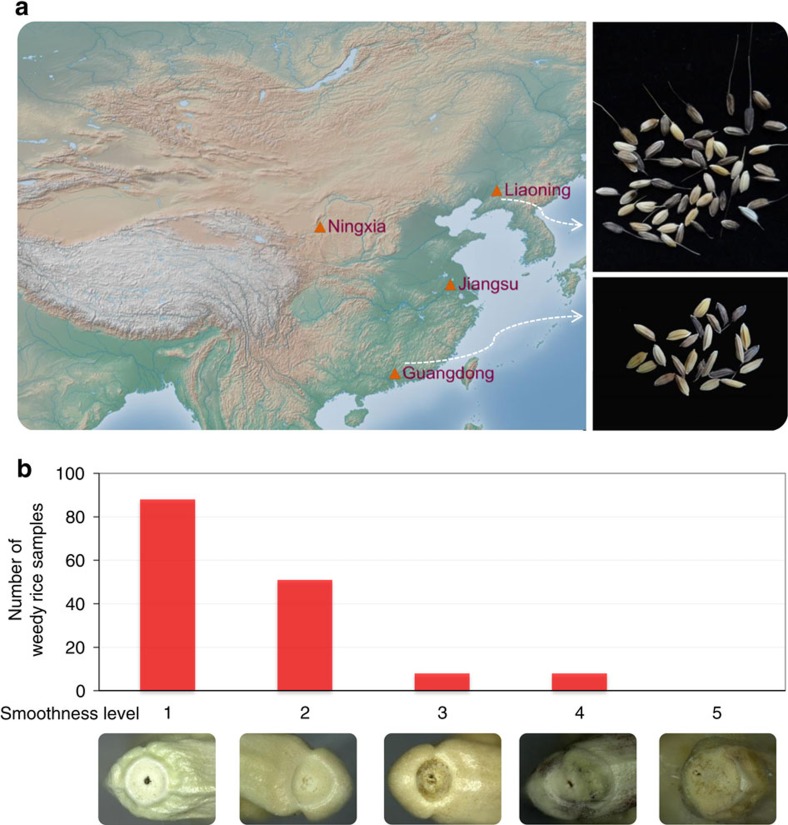
Geographic locations and phenotypes of Chinese weedy rice used in this study. (**a**) Geographic locations of the four provinces where weedy rice were collected in the left map (map courtesy of Natural Earth), whereas seeds of weedy rice from Liaoning and Guangdong provinces are shown on the right. (**b**) Smoothness levels of spikelet bases of rice samples. Top, smoothness levels were scored from 1 to 5 (highly smooth to rough). Bottom, an example of a spikelet base from each Smoothness level category.

In order to generate a representative population of Chinese weedy rice isolates, 155 samples were collected from multiple fields in four heavily infested provinces in China (Supplementary Data 1; another seven weedy rice were collected from United States and South Korea). The four areas include typical *indica* rice production areas in south China (Guandong), *japonica* rice growing areas in northern China (Liaoning and Ningxia) and mixed *indica*–*japonica* production areas in the Yangtze regions (Jiangsu) (Fig. 1a). As comparative partners, we also collected 76 cultivars that are historically grown in these same four provinces. In contrast to the cultivated rice samples collected in this study, the majority of weedy rice samples collected have a seed shattering phenotype and brown seed coat (Table 1; Fig. 1b). The extent or rate of shattering in the various weedy rice samples was estimated based upon the smoothness of their spikelet bases, which is an indication of the maturity level of the abscission layer that is responsible for the shattering phenotype^22^,^23^. In general, we found that the spikelet bases in the weedy rice bases were smoother (mean=1.6) compared with those of cultivated rice (mean=3.9), and most of the weedy rice forms (89.7%) had a smooth spikelet phenotype (1–2) (Fig. 1). Among the weedy rice isolates 42.6% had dark hulls and 43.9% had awns (Table 1).

All 238 accessions (162 weedy and 76 local cultivated rice) were sequenced and a total of 1,615 Gb of raw data (in average ∼18.2 × depth) were generated for the collected samples (weedy: 1,074 G; cultivated: 541 G). Cleaned reads of each accession were mapped to the *japonica* rice reference genome (MSU v6.1), and ∼93.4% of the reference genome was covered per sample. Genomic data from a representative collection of 160 *Oryza* species sequenced by Huang *et al*.^15^ were integrated in this study (for details see Materials and Methods) to form a combined genotype table for the 398 accessions. An imputation approach was applied to predict the missing genotypes^24^. We also sequenced one gene (*OsEXP3*) in 38 accessions of the Jiangsu *indica* weedy group using Sanger-based methods in order to determine the genotype at five single-nucleotide polymorphisms (SNP) locations (see Materials and Methods). These analyses found that all the genotypes were consistent between the two methods if heterozygous genotypes were not considered (Supplementary Table 1). Finally, a total of 7,782,704 SNPs were genotyped based on the 398 accession set and the combined SNP genotype table was used in our subsequent analysis.

### The origin of weedy rice

**Figure 2:**
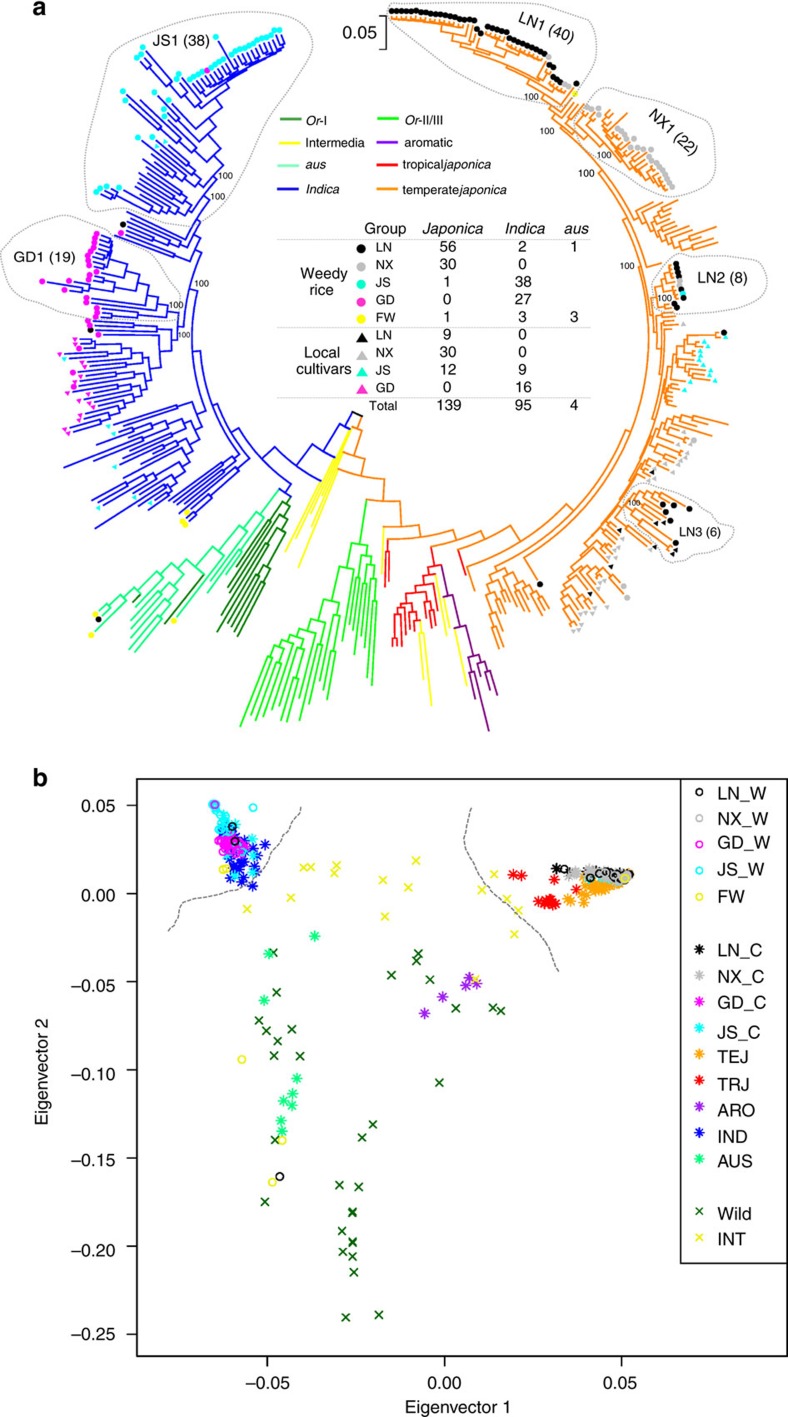
Population structure analysis of weedy rice based on genome-wide SNPs. (**a**) A maximum-likelihood phylogenetic tree of weedy and cultivated rice with other *Oryza* species. Different subgroups are coded with different colours: *O. rufipogon*: green for the *Or*-II and *Or*-III subgroups, dark green for the *Or*-I subgroup; *aus*: light green; *O. sativa* ssp. *japonica*: purple for aromatic *japonica* (ARO), red for tropical *japonica* (TRJ) and orange for temperate *japonica* (TEJ); *O. sativa* ssp. *indica* (IND): blue; intermediate type: yellow. The symbols for weedy and local cultivars from different geographical locations (LN for Liaoning, NX for Ningxia, JS for Jiangsu, GD for Guangdong and FW for weedy rice sampled outside of China) are illustrated in the middle of the phylogenetic tree. Six main de-domestication origins (LN1, LN2, LN3, NX1, JS1, GD1) for weedy rice from four Chinese provinces are marked within the dotted circle. The number of accessions of each origin is shown in paratheses. Node support is indicated by bootstrap values. (**b**) Principle component analysis (PCA) plot of weedy and cultivated rice with other *Oryza* species by the first and second eigenvectors. All weedy rice are marked by ‘o' while cultivated rice by ‘ × ' and wild-/intermediate-type rice by ‘*'. In the legend, the suffix ‘W' refer to weedy rice, and ‘C' for local cultivated rice. Two grey lines were used to separate *indica* and *japonica* subspecies from others.

Huang *et al*.^15^ used 1,529 *Oryza* lines to construct a comprehensive phylogenetic tree of the rice genus. In this study, we selected 160 representative lines encompassing the major branches on the rice phylogenetic tree and were able to construct a phylogenetic tree with the similar topology as reported by Huang *et al*.^15^. Then we used the genome-wide SNPs from the 238 weedy/local cultivated rice examined in this study together with the 160 genetically defined rice accessions to construct a new phylogenetic tree (Fig. 2a; Supplementary Fig. 1). The 238 lines integrated into the diverse branches of the tree without significantly altering its general topology. As shown, both the *indica* and *japonica* forms of *O. sativa* were completely separated and descended from wild rice *Or*-I and *Or*-III groups, respectively. Based on the tree, we found that: (1) all Chinese weedy rice clustered into a cultivated rice group and a number of them near their historical cultivated rice collected from the same province; (2) the majority of weedy rice lines (86/89) collected from the Liaoning and Ningxia of northern China, where *japonica* rice is grown, were clustered into the *japonica* rice clade, whereas only two and one lines from Liaoning province were grouped into *indica* and *aus* rice clade, respectively; all weedy rice sampled from Guangdong in south China where *indica* rice is grown clustered into the *indica* rice clade; weedy rice from Jiangsu regions, the mixed *indica–japonica* rice production of China, were clustered into the *indica* rice clade with only one sample falling into the *japonica* rice clade; (3) the Korean weedy rice (FW_8) resided in the *japonica* rice clade and shared a very close relationship with Liaoning weedy rice (for example, LN_W63); of the six weedy rice samples collected from the USA, three grouped into the *indica* clade and three grouped with *aus* rice consistent with the previous report by Reagon *et al*.^9^; (4) There were likely at least six independent origins for our sampled Chinese weedy rice, three for Liaoning (LN1, LN2, LN3) and one for each of three other provinces (NX1, JS1, GD1). The bootstrap confidence values for the topology of all the six clades are 100% (Fig. 2a). Although LN1 and NX1 appear together as a monophyletic group and more data may be needed to conclusively determine their origin, they are from geographically distinct regions with distinct climates and environments, and are thus treated separately for population genetic analysis.

Principle component analysis (PCA) based on the genome-wide SNPs confirmed the population structure evidenced in the phylogenetic tree (Fig. 2b). In the two-dimension PCA plot based on the first two eigenvectors, *indica* and *japonica* cultivated rice were clearly separated from intermediate rice (as described by Huang *et al*.^15^) and wild rice. The PCA also supports our phylogenetic analysis indicating that all Chinese weedy rice cluster with the cultivated rice forms. Consistent with previous reports^10^,^12^,^13^, our results collectively showed that none of the Chinese weedy rice sampled in this study directly originated from wild rice types, and the weedy forms collected in this study originated by multiple independent de-domestication events, most likely from the historically locally grown cultivars. Based on our phylogenetic analysis, four subpopulations (LN1, NX1, GD1 and JS1) that each likely represents a main single de-domestication event of one province were used for subsequent population genetic analyses (Fig. 2a). The samples size for the four sub-populations were 40, 22, 19 and 38 for LN1, NX1, GD1 and JS1, respectively. Some other minor origins, including the two de-domestication subgroups of Liaoning (LN2, LN3), were not considered for further analyses owing to limited sample size.

### Genetic bottleneck and demographic inference of weedy rice

**Figure 3:**
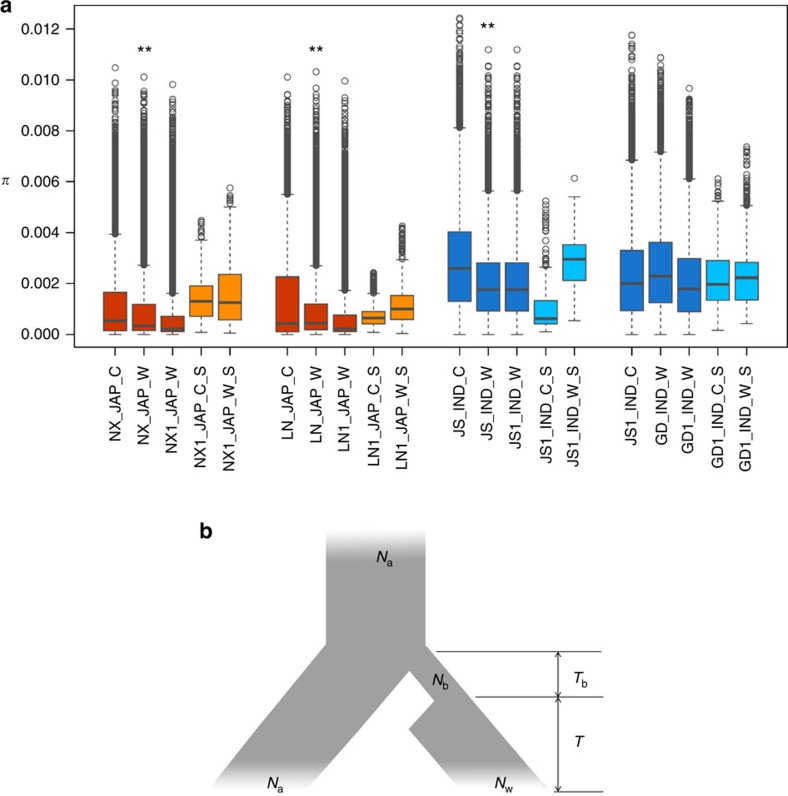
Genetic diversity and demographic model for weedy rice. (**a**) Boxplots of genetic diversity (*π*) for weedy and cultivated rice. Boxes with orange colour are *japonica* type (JAP), and blue for *indica* type (IND). Cultivated rice is labeled with ‘_C', whereas weedy rice with ‘_W'. Genetic diversity for significant divergent windows of weedy from cultivated rice are marked in light orange or blue colour with the suffix ‘_S'. The prefixes ‘NX', ‘LN', ‘JS', ‘GD' are short for Ningxia, Liaoning, Jiangsu and Guangdong provinces, respectively. The prefixes ‘NX1', ‘LN1', ‘JS1', ‘GD1' are short for main de-domestication origin of Ningxia, Liaoning, Jiangsu and Guangdong provinces, respectively. The ‘**' is marked if the genetic diversity of weedy rice is significantly lower than that of local cultivated rice (*P*<0.01 by paired *t*-test). (**b**) Demographic model for weedy rice. The model assumes that the initiate weedy rice founders split from cultivated rice (effective population size *N*_a_) and then suffered a genetic bottleneck in a *T*_b_ duration. After the bottleneck, the weedy rice founder population (effective population size *N*_b_) recovered to the present effective population size (*N*_w_) at time *T*.

We used nucleotide diversity (*π*) to measure the genome-wide genetic diversity of the Chinese weedy rice populations (Fig. 3a). Our data showed the distribution of genome-wide diversity was significantly lower for weedy rice collected from three provinces than that of their counterparts in cultivated rice (*P*<0.01 by paired *t*-test). However, we also noted that the genetic diversity of Guangdong weedy rice is higher than that of its local cultivated rice. Based on the phylogenetic tree (Fig. 2a), we could assure that Guangdong weedy rice not directly originated from wild rice, but originated from cultivated rice. Therefore, we proposed that the higher diversity of Guangdong weedy rice is possibly owing to wild rice introgression^11^, as Guangdong province locates in South China where many wild rice are grown. A genetic bottleneck model was proposed for the origin of weedy rice in the United States^9^. We then tested the origin models of Chinese weedy rice using four different demographic simulation models (Supplementary Data 2; Supplementary Fig. 2). Based on SNPs in intergenic regions or at fourfold degenerated sites (putative neutral variants), we found that the bottleneck model (Fig. 3b) provided a better fit to the data (Supplementary Data 2), supporting the conclusion that both the *japonica* (LN1 or NX1) and *indica* (JS1 or GD1) weedy rice types suffered a genetic bottleneck effect during their split from their progenitor cultivated forms. We also estimated the timing of de-domestication event for each Chinese weedy rice group from its cultivated progenitor. We found that the split time of weedy rice of four provinces was different but fell within the same general time span of 300∼1,400 years ago (Supplementary Data 2). These estimates are consistent with written records about weedy rice in ancient Chinese agronomy books, which indicate that Chinese weedy rice, such as ‘Ludao', could be traced back to the Song Dynasty (A.D 960–1,279)^25^. Moreover, interestingly, unlike cultivated rice that experienced a long bottleneck period of ∼3,000 years^26^, weedy rice suffered from a short bottleneck duration of less than 150 years during de-domestication. In addition, it appears that only a very small number of weedy rice individuals (usually <100) survived from the bottleneck and became the founding population, which is much smaller than the founding population number of cultivated rice (estimated at 200–500 individuals)^27^ (Supplementary Data 2).

### Genomic differentiation during rice de-domestication

**Figure 4:**
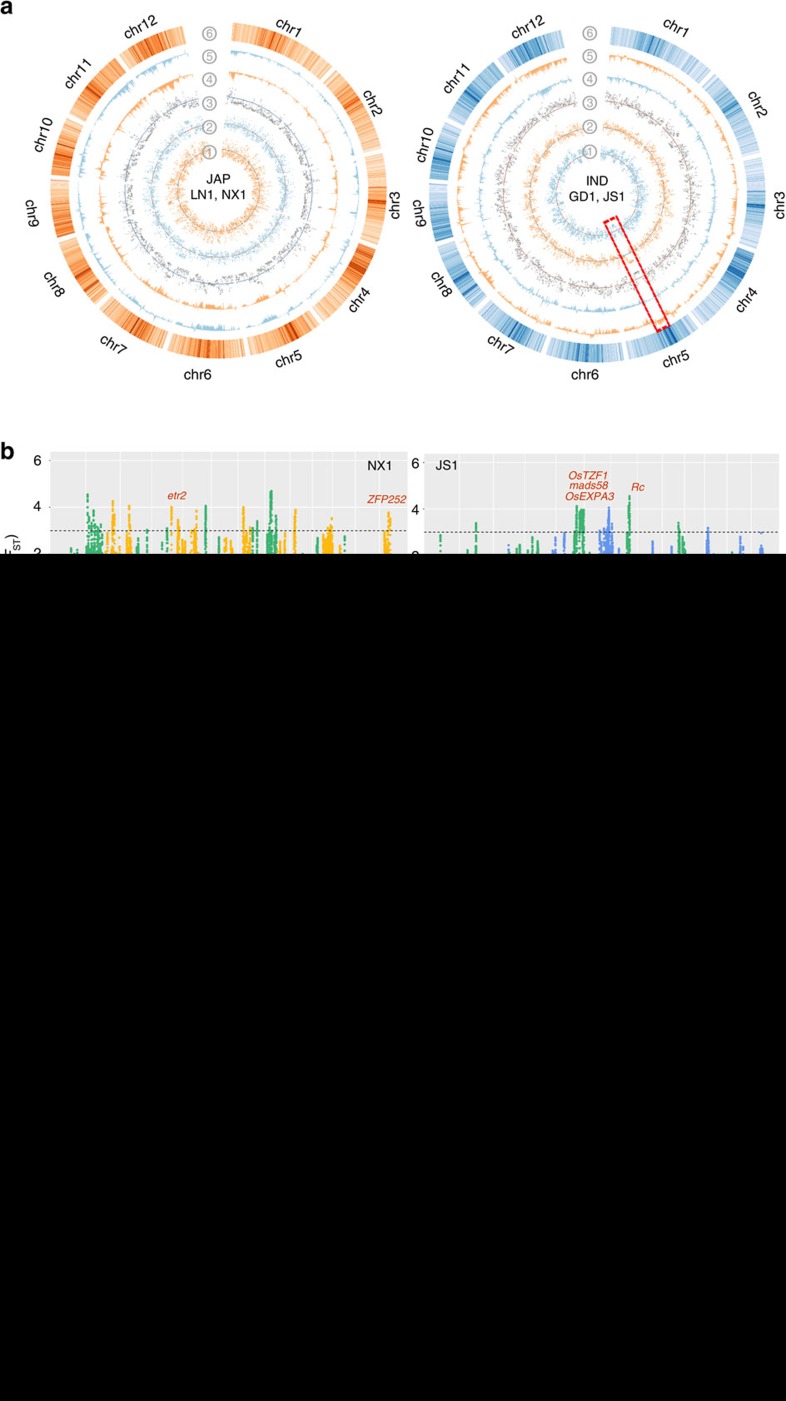
Genomic differentiation and selection between weedy and local cultivated rice. (**a**) Genomic distribution of population summary statistics of *japonica* (left) and *indica* type (right) weedy and local cultivated rice. The population parameters (*π*, Tajima's *D*) in this study were estimated in a 100 Kb window size along the reference genome. Legends for circles from inside to outside: Tajima's *D* of the LN1 (left) and GD1 (right) Tajima's *D* of NX1 (left) and JS1 (right); Tajima's *D* of TEJ (left) and IND (right); *π*_JAP_*−π*_LN1_ (left) and *π*_IND_*−π*_GD1_ (right), with inward present positive value; *π*_JAP_*−π*_NX1_ and *π*_IND_*−π*_JS1_ with inward present positive value; repeat density across the reference genome. (**b**) *F*_ST_ between weedy and cultivated rice from different locations. *Japonica* weedy rice from Ningxia (NX1) and Liaoning (LN1) and *indica* weedy rice from Jiangsu (JS1) and Guangdong (GD1) were illustrated, respectively. Some functionally known genes locating the significant divergent regions were shown. (**c**) A common genomic region with significant divergence in both *indica* and *japonica* weedy rice populations relative to their cultivated rice. The domestication gene *Rc* controlling pericarp colour and dormancy and a cluster of seed allergenic genes (*RAL*) locate in the region.

Genome re-sequencing allowed us to perform large-scale genomic scans to uncover whole genomic changes after rice de-domestication (Fig. 4a,b). Using *Z*(*F*_ST_) to measure genomic differentiation of four subpopulations^28^, we detected significant differentiation regions across the genome between weedy and cultivated populations (Fig. 4b). With a threshold of *Z*(*F*_ST_)≥3.0, a total of 505 and 746 divergent windows containing 1,031 and 929 genes were identified in Ningxia and Liaoning main de-domestication subpopulations, respectively; whereas for Guangdong and Jiangsu, we identified 767 and 644 divergent windows with 1,428 and 709 genes resided in. In general, many important functional genes are among the candidates associated with de-domestication (Supplementary Data 3). For example, among these genes are *sh4* and *sh-h* involved in seed shattering^29^,^30^, *Rc* controlling the rice pericarp colour^31^, *OsMADS51* (ref. 32) and *Ehd4* (ref. 33) responsible for controlling flowering time, and *msp1* (ref. 34), *OsMS1* (ref. 35) involved in reproduction. In addition, we also detected several genes involved in disease/pest resistance and stress-tolerance, such as the salt/drought tolerance genes *OsEXPA3* (ref. 36) and *ZFP252* (ref. 37), blast resistance-related gene *WRKY30* (ref. 38) (Fig. 4b; Supplementary Data 3). However, very few of the divergent genes or regions (an example in next paragraph) were shared between different groups. For *japonica* type, only 134 genes were shared between Ningxia and Liaoning, whereas 84 were in common in the *indica* type from Jiangsu and Guangdong (Supplementary Fig. 2). We further checked whether the identified differentiation genes between weedy and cultivated rice resided in the domestication related regions^15^. However, the overlapping rate for each province was very low, ranging from 4.0 to 23.0% (Supplementary Fig. 3). Our results indicated that during de-domestication, different weedy rice types underwent local adaptation in which different subsets of adaptive genes were selected for in response to various selective pressures present in the different environments where the weedy rice was growing. The little overlap rate between loci associated with domestication and de-domestication may suggest that weedy rice can employ other adaptive genes or loci rather than simply reversing domesticated genes to their wild types.

It was also possible for us to determine whether there are any shared genomic differentiation ‘hotspots' in the various weedy rice populations during de-domestication from cultivated rice. As shown in the Venn diagram in Supplementary Fig. 3, 15 genes were found to be common differentiated among at least three groups. The 15 genes are located in adjacent genomic regions (6.0–6.4 Mb of chromosome 7; Supplementary Data 4). We also observed that the gene ontology (GO) enrichment terms for either Guangdong and Jiangsu weedy rice were similar, showing significant categories like ‘adaptive immune response' (GO:0002250), ‘hypersensitivity' (GO: 0002524), ‘oxidation reduction' (GO:0055114) and ‘response to external stimulus' (GO:0009605) (Supplementary Data 5). Intriguingly, these enriched terms are due to a cluster of seed allergenic genes containing the same Pfam ‘PF00234' (Protease inhibitor/seed storage/LTP family), which are successively arranged in the shared highly divergent region of the 0.4 Mb regions on chromosome 7 (Fig. 4c). Notably, *Rc* is also in that region and adjacent to the gene cluster. These results suggest that the genomic region covering the seed allergenic gene cluster and *Rc* may be indispensable for rice de-domestication. Thus we would expect that these genes in different weedy populations may be under intense natural selection and the product of convergent evolution for similar critical functions.

### Roles of standing variations and new mutations

**Figure 5:**
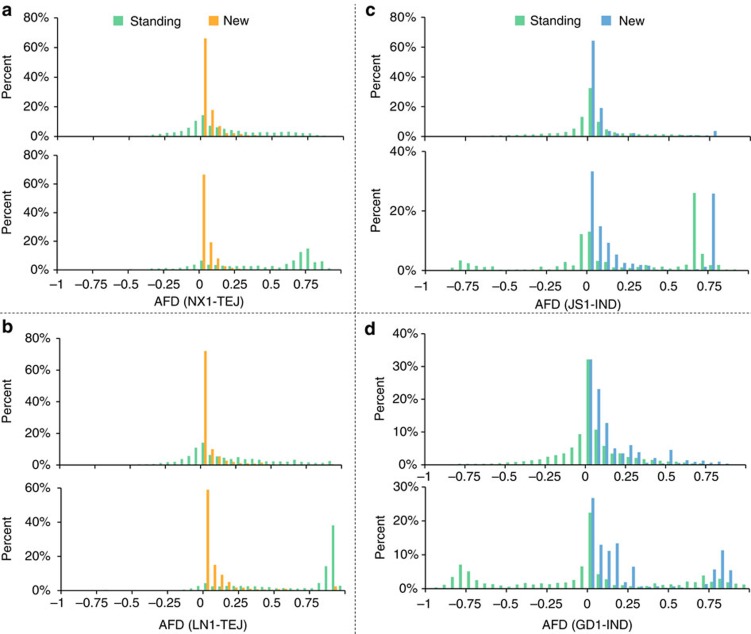
Percentage distribution of alternative allele frequency differentiation for standing and new variations between weedy and cultivated rice. The AFD was measured by subtracting the alternative allele frequency of cultivated rice group from the corresponding weedy rice. (**a**) NX1-TEJ; (**b**) LN1–TEJ; (**c**) JS1–IND; (**d**) GD1–IND. The distributions for genome wide SNPs were shown on the top of each subfigure, and SNPs in the identified selected regions (Fig. 4b) are shown at the bottom. Percentages of standing variations are plotted in green histograms, and new mutations of *japonica* and *indica* type are depicted in orange and blue histograms, respectively.

Based on the genome-wide identified SNPs and population classification, we found most SNPs of weedy rice (NX1: 95.7%; LN1: 92.3%; JS1: 97.2%; GD1: 97.9%) were shared with cultivated rice. This suggested that the majority of variations of weedy rice were pre-existing or standing and likely originating from cultivated rice (Supplementary Fig. 4). We further explored the extent to which the allele frequency of standing variations and putatively new mutations differed in each of the four weedy rice populations compared with its progenitor population, separately. Using cultivated rice (Nipponbare) as the reference genome and the cultivated rice population as background, we calculated the alternative allele frequency differentiation (AFD) between weedy and cultivated rice for each SNP in the whole weedy rice genome and also the identified selected regions. With a threshold of AFD>0.7, we found a much higher percentage of SNPs with high AFD for standing variations in comparison to new mutations for *japonica* type weedy rice (LN1 or NX1). However, a different trend was observed for *indica* type (GD1 or JS1). When the threshold of AFD was set above 0.7, we found the percentage of new mutations that have high AFD is unexpectedly greater than that of standing variations, but standing variations still have higher percentage of SNPs with AFD>0.9 (Fig. 5; Supplementary Data 6; Supplementary Data 7). These findings suggest that standing variations have a more rapid allele fixation rate than new mutations during de-domestication, and thus may have a critical role for rapid adaptation. In addition, variations that are not pre-existing in cultivated rice also contribute greatly to the adaptation of weedy rice. This appears to be particularly true for Chinese *indica* type weedy rice.

### A Mb-scale genomic region under balancing selection

We measured nucleotide diversity (*π*) in the identified divergent regions between weedy and cultivated rice. Somewhat unexpectedly we found a generally increased rather than decreased genetic diversity in weedy rice populations compared with cultivated rice (Fig. 3a; Supplementary Table 2). The results do not support the occurrence of a significant genetic bottleneck in weedy rice (that is, generally the lower genetic diversity of weedy rice than their local cultivated rice as shown in above section) and imply that some driving forces other than positive selection or demographic effect may have a crucial role during the de-domestication process.

We performed a Tajima's *D* measurement for each weedy rice subpopulation across the genome to identify potential type of selection during rice de-domestication process (Fig. 4a). Several large genomic regions (above 0.5 Mb in length) in weedy rice populations had significantly high Tajima's *D* values (top 5% across the genome) but relatively low values (*D*<1) were found in the corresponding cultivated rice (Supplementary Table 3), which indicated that potential recent balancing selection might act on these genomic regions of different weedy rice groups during de-domestication. Interestingly, we found a 4.4 Mb region located on chromosome 5 of Jiangsu subpopulation (JS1) having apparent higher Tajima's *D* values (2.9, on average of the sliding genomic windows), but very low *D* values (−1.8) were observed in all *indica* cultivated rice (highlighted in Fig. 4a; Table 2). We also measured the observed heterozygosity (*H*_*O*_) per SNP in the region for JS1 and *indica* rice, and found that the weedy rice (*H*_*O*_=0.052) had significantly higher heterozygosity in average than the corresponding cultivated rice cultivars (*H*_*O*_=0.010) in that region (*P*-value=2.2e-16, by paired *t*-test). In addition, the region reside in top 5% high Tajima's *D* windows of JS1 and significant differentiation genomic regions compare to cultivated rice (*Z*(*F*_ST_)≥3), suggesting a low possibility of bias owing to genetic drift. The region is located in a relatively repeat-rich portion of the chromosome and covered the centromeric area of the chromosome (Fig. 4a). To confirm novel genomic structure in the region was not caused by repetitive elements, we calculated the Tajima's *D* of non-transposable element (non-TE) genes within the region and we consistently observed much higher numbers of non-TE genes with Tajima's *D*>2 in the region of JS1 subpopulation but low in cultivated rice (Table 2; Supplementary Data 8). Furthermore, we also sequenced a abiotic stress-related gene (*OsEXPA3*) in the gene set in the Jiangsu *indica* weedy rice populations by Sanger approach (Supplementary Fig. 5) and confirmed a significant high Tajima's *D* for this gene in JS1 weedy rice (*P*-value=0.029, tested by 10,000 coalescent simulations). Taken together, we believe that the Mb-scale regions of Jiangsu weedy rice experienced strong balancing selection since their de-domestication from cultivated rice populations.

## Discussion

The genome-based study carried out here indicates that Chinese weedy rice were de-domesticated from cultivated rice and are a good model to uncover signature changes in genome architecture associated with crop de-domestication. To our knowledge, this is the first attempt to examine crop de-domestication at whole genomic level. Our findings not only facilitate a better understanding of this unique evolutionary process, but also have practical implications for paddy weed management.

Many long-standing questions regarding rapid adaptation in plants remain to be answered^39^,^40^. One of the issues is the relative roles standing (pre-existing) variations and newly arisen mutations play for environmental adaptation during de-domestication. The roles of standing and new variations in the de-domestication system were investigated in this study from a whole genomic view. We found a much more rapid allele frequency change rate for standing variations, suggesting the standing variations play important role in local adaption of weedy rice. Previous studies have suggested that standing variation could facilitate more rapid adaptation than through new mutations, because an initially higher frequency of beneficial alleles is immediately available for standing variation, which reduces the average fixation time^40^. On the other hand, we observed that many SNPs that were not pre-existing in the progenitor cultivated rice also have a high allele frequency in weedy rice, which is particularly the case for *indica* type weedy rice. The new mutations or variations introgressed from wild rice^11^ should also contribute to play critical roles and provide a sustainable supply to the species adaptation during de-domestication process.

Another question is the selection regime involved in the de-domestication process. For domestication, the rapid evolution of crops is mainly driven by human-imposed directional selection. However, de-domesticated plants are subject to more diverse pressures and may require more capabilities to survive in an agro-ecosystem, such as the capacity to escape from human weeding, to persist in the seed bank, and to compete for light and nutrients with crops nearby. In our genome-wide investigation, some genomic regions were found to have undergone potential balancing selection. However, most of them were only observed in one subpopulation, which is consistent with the fewer overlapping results of the numbers of selected genes. We believe it is also due to local adaptation, during which different subsets of loci were targets of balancing selection in response to various selective pressures present in the different environment where the weedy rice was growing. A Mb-scale region was found to be under balancing selection. This region resides near the centromere where re-combination is relatively rare. This may in part explain the large width of the signature. The region also has significantly higher heterozygosity compared with its counterpart in cultivated rice type indicating that heterozygous advantage could be one of the mechanisms for the balancing selection in weedy rice. On the other hand, the Tajima's *D* values of these regions in cultivated rice were very low, indicating alleles with low frequency in cultivated rice evolved to medium frequency in weedy rice during de-domestication. Therefore, the mechanism of negative frequency dependence could not be neglected. Prior to our analysis, the best-known examples of genes under balancing selection in plants were those involved in self-incompatibility and pathogen resistance^41^. Several genes related to resistance and self-incompatibility reside in these regions, including the salt tolerance gene *OsEXPA3* and the reproduction related genes, ‘LOC_Os05g18730' and ‘LOC_Os05g18940' containing the domains ‘Male gamete fusion factor' and ‘Stigma-specific protein, *Stig1*', respectively (Supplementary Data 8). These genes have very high Tajima's *D* in weedy rice but quite low values in its progenitor cultivated population indicating strong balancing selection. Craig *et al*.^42^ have reported that genetically based postzygotic barriers to hybridisation between weedy and cultivated rice are not strong, and they indicated a greater potential than expected for crop-weed hybridisation in US cultivated rice fields. Consistently, adaptive introgression from local cultivated or wild rice has played a critical role in the adaptation of weedy rice^11^,^19^. Balancing selection would therefore be expected to help maintain a high polymorphism in the adaptation-related genomic regions and provide an evolutionary mechanism for adaptations during crop de-domestication.

During de-domestication, the genomic pattern under balancing selection is quite distinct to what is observed in crop domestication, which is usually with a reduction of genetic diversity by artificial selection. Based on our whole genomic analyses, many regions under genomic differentiation have evolved to have a higher genetic diversity and heterozygosity relative to their progenitor cultivated rice during de-domestication (Fig. 3a). Within these regions are located many genes that may be important in environmental adaptation including genes controlling flowering time, reproduction, disease/pest resistance, and other stress responses (Supplementary Data 3). This could help explain part of the extensive morphological diversity that exists in weedy rice populations despite their generally lower genome-wide genetic diversity^9^. The observed phenotypic heterogeneity, such as multiple flowering strategies, can be regarded as an indicator of better adaptive flexibility^11^,^43^. As weedy rice likely diverged from cultivated rice hundreds of years ago, the two rice types are under distinct selection pressures driving their genomes to evolve. The genomic differentiation scan between weedy and modern cultivated rice may be compounded by both artificial selection and natural selections. In our study, 4–23% of de-domestication genes overlapped with the regions under artificial selection. We believe that some of these genes or loci probably crucial for both domestication and de-domestication (for example, *Rc*). However, there could also be some genomic differentiation regions between weedy and cultivated rice only due to artificial selection on cultivated rice. Therefore, it is indeed difficult to clearly separate the complex effects of the two genetic processes in those overlapping regions.

This study also provides some practical implications for the paddy weed control. Our analyses indicate that different weedy rice populations have independent origins and employ different genomic loci for their adaptation and weediness traits. Therefore, it may be not easy to develop a universally efficient weedy rice management approach. More likely different strategies will be needed to tackle the problems of weedy rice in different regions. Rice breeders have long sought after a greater genetic resource pool to incorporate in their genetic improvement work. As evidenced in this study, genetic diversity of some important environmental adaptation genes has increased in weedy rice driven by balancing selection during de-domestication. Thus, locally generated weedy rice could supply a valuable genetic resource for fitness genes to local environments, contributing to the more rapid improvement of tolerance and resistance traits in cultivated rice.

## Methods

### Plant materials and DNA sequencing

A total of 155 weedy and 76 local cultivated rice samples were collected from China and with seven weedy samples from the USA and South Korea used in this study (Supplementary Data 1). The 155 Chinese weedy rice accessions were collected from multiple rice planting fields of four representative geographically locations or provinces in China (Liaoning, Ningxia, Jiangsu and Guangdong) where rice fields are seriously harmed by weedy rice. The geographic map was plotted using GenGIS v2 (ref. 44). The 76 cultivated rice including local landraces and cultivars planted in recent 30 years in the four regions. Liaoning and Ningxia locate in northern China which temperate *japonica* is widely cultivated, whereas Guangdong in Southern China primarily cultivates *indica* rice. Jiangsu province locates in the low Yangtze region, where both *indica* and *japonica* rice types were cultivated in history, and in recent 20 years, *japonica* varieties have become the dominant type. After collecting the seeds of weedy and cultivated rice accessions, they were stored in China National Rice Research Institute. Seeds were germinated and planted together in the experiment field (Chinese Academy of Agricultural Sciences in Fuyang, China). The seeds of each accession were harvested when grains ripened (∼30 days after flowering) to minimize bias owing to different seed maturity level. Five seed traits (shattering, presence of awn, pericarp colour, hull colour, seed length width ratio) were evaluated. The extent of shattering was estimated based on the smoothness of their spikelet bases, which is an indication of the maturity level of the abscission layer that is responsible for the shattering phenotype^22^,^23^. The entire DNAs of the 238 samples were extracted from green leaves using routine protocol. A total 1,615 Gb paired-end sequence data were generated by Illumina Hiseq2500 and Hiseq4000, covering approximately an average depth of 18.2 × for each sample. The short-read sequence data by this study have been deposited into the GenBank under the bioproject accession number PRJNA295802.

Other genomic data from 160 *Oryza* species (including 34 *indica* varities; 50 and 14 temperate and tropical *japonica* varities; 5 aromatic; 32 *O*. *rufipogon*; 9 *aus* and 16 intermediate accessions) (Supplementary Data 1) were obtained from previous study^15^.

### Variants detection and genotyping

The raw paired-end reads were first filtered into clean data using NGSQCtookit v2.3.3 (ref. 45). The cutoff value for PHRED quality score was set to 20 and the percentage of read length that met the given quality was 70. Clean reads of each accession were mapped to *japonica* rice reference genome MSU v6.1 (ftp://ftp.plantbiology.msu.edu/pub/data/Eukaryotic_Projects/o_sativa/annotation_dbs/pseudomolecules/version_6.1/all.dir/) using BOWTIE2 v2.2.1 (ref. 46) with default settings. Consecutive steps using Samtools v0.1.19 (ref. 47) and GATK v2.3 (ref. 48) were applied for variants detection. Potential PCR duplicates were removed by ‘Samtools rmdup'. Alignments around small indels were remapped with ‘IndelRealigner', and raw variants were called based on the realigned bam file. Using the called variants as known sites, ‘BaseRecalibrator' and ‘PrintReads' in the GATK were applied for base-pair scores recalibration. The proceeded BAM files of each sample were used for the multi-sample variant genotyping. ‘UnifiedGenotyper' in GATK was applied to generate the raw variant calls with parameters ‘-stand_call_conf 30, -stand_emit_conf 10'. To reduce the variants discovery rate, the SNP calls were filtered according to the following threshold: QUAL<30, DP<5, QD<2, MQ<20, FS>60, HaplotypeScore>13 and ReadPosRankSum<−8. Potential variant annotation and effect were predicted by SnpEff v3.6 (ref. 49). Imputation was performed by BEAGLE v4.0 (ref. 24) using the genotype likelihoods, and specified the number of iterations for estimation genotypes at genotyped markers to 10.

### SNP validation by Sanger sequencing

For genotyping validation, approximately an 850 bp portion of one gene (LOC_Os05g19570, *OsEXPA3*) in the potential balancing selection Mega-base region was selected to sequence by Sanger method in all 38 Jiangsu *indica* type weedy rice sampled in this study. The PCR-amplified segment includes 5 SNPs based on our next generation sequencing SNP calling. The Primers were listed in Supplementary Table 1.

### Population structure inference

Based on the genome-wide SNPs among the 238 lines by this study and the 160 public samples^15^, phylogenetic tree was constructed using Fasttree^50^ with 1,000 replicates for bootstrap confidence analysis. MEGA v5.1 (ref. 51) was applied to draw the constructed tree. PCA was performed by SNPRelate v0.9.19 (ref. 52).

### Demographic analysis

To minimize bias in demographic analyses due to selection, SNPs in intergenic regions or at four-fold degenerate sites were used. The best parameters for fitting model were estimated by ∂a∂i v1.6.3 (ref. 53). Four weedy rice sub-populations were independently subjected to demographic inferences (that is, LN1, NX1 de-domesticated from TEJ, respectively; JS1, GD1 de-domesticated from IND, respectively). The alleles were downsampled via hypergeometric projection for each group (LN1: 70; NX1: 40; GD1: 36; JS1: 70; TEJ: 100; IND: 70). Folded spectrum was used and singletons were masked for each pool. Four demographic models were considered for each type: weedy rice split from cultivated rice with bottleneck, and can/cannot migrate with cultivated rice after bottleneck duration; weedy rice split from cultivated rice without bottleneck and allow/does not allow migration (Supplementary Fig. 2). Different demographic models were compared on the basis of the relative log likelihoods of the models given the observed site frequency spectrum. Two hundred independent runs with randomized starting points were executed for each candidate model, and median value was chosen based on the best fitting parameters. Among the inferred parameters, the population size was scaled by *Ne* (ancestral effective population size), whereas migration rate and time was scaled by 2*Ne* (ref. 53). The ancestral population size was estimated for *indica* type and temperate *japonica* type by the formula *θ*=4 × *Ne* × *μ* × *L*, where the genetic diversity (*θ*) is set to 2.2e-3 and 1e-3 for *indica* and temperate *japonica* cultivars according to Caicedo *et al*.^54^, the neutral mutation rate (*μ*) is set to be 6.5e-9 (ref. 55), and the generation time (*L*) was assumed to be 1 year. Therefore, *Ne* was estimated as 84,615 and 38,461 for *indica* and temperate *japonica* type, respectively.

### Population parameters estimation

The genome was scanned in a 100 Kb window size and the population parameters (*π*, *F*_ST_, Tajima's *D* and heterozygosity rate) were estimated for each window by VCFtools^56^. Nucleotide diversity (*π*) was measured with parameters ‘--window-pi 100,000 --window-pi-step 10,000'. The average 100 k window size π value was taken as the genetic diversity. For measurement of population differentiation, *F*_ST_ was calculated with the setting ‘--fst-window-size 100,000 --fst-window-step 10,000'. Heterozygosity rate was measured by the *H*_*O*_ for each SNP by an in-house script. The parameters across the genome were plotted by Circos v0.62 (ref. 57). To minimize the bias for identification of genes under balancing selection, we further performed Tajima's *D* calculation for all genes in the reference genome (MSU v6.1) using PopGenome v2.16 (ref. 58).

Summary statistics for the portions of the gene (*OsASR3*) sequenced by Sanger method were performed by DnaSP v5 (ref. 59). Significance was tested with 10,000 coalescent simulations.

### Standing and new variations analysis

As weedy rice derived from cultivated rice, we can generally classify the SNPs of weedy rice into pre-existing SNPs originated from cultivated rice and new SNPs raised during the de-domestication process. Here, the shared SNPs between them were regarded as standing variations, whereas unique SNPs of weedy compared with cultivated rice were taken as new mutations. The numbers of the standing and new variations of weedy rice from four regions (NX1, LN1, JS1 and GD1) were calculated separately. All wild rice accessions (including *Or*-I, *Or*-II and *Or*-III) were taken as the wild pool. We estimated the allele frequency differing rate of standing and new SNPs between weedy and cultivated rice by AFD^16^. Given the genome reference is cultivated rice (Nipponbare), we first calculated alternative allele frequency of each SNP for each rice group, and calculated AFD between weedy and its counterpart cultivated rice by alternative allele frequency (weedy)—alternative allele frequency (cultivated). The percentages of SNPs (across the whole genome and within the selected regions) with high AFD (≥0.7) were calculated for each weedy rice group.

### Genomic differentiations detection

In the genomic differentiation analyses based on population differentiation index (*F*_ST_), we compared weedy rice of one province against the totality of its potential ancestors (for example,, LN1 versus all *japonica* cultivars). *Z*-transformation was applied to locate divergent regions between weedy rice and cultivated rice from the extreme tails by applying a threshold of three s.d.s^28^ The non-reductant genes residing in these regions were taken as putatively selected de-domestication genes. To examine whether the divergent genes between weedy and cultivated rice residing in the domesticated regions, we mapped our identified de-domestication related genes to the rice reference genome (IRGSP Build 4.0) used by Huang *et al*.^15^, and checked the percentage of genes overlapping with the domestication-related regions (for *japonica* and *indica*, separately) identified by them^15^. For the detection for genomic regions with potential balancing selection for weedy rice, the genomic windows with top 5% high Tajima's *D* were found for each weedy rice group, that is, Tajima's *D* value threshold for JS1, GD1, LN1 and NX1 were 2.46, 2.55, 3.09 and 2.50, respectively. Large genomic regions (>0.5 Mb) with continuous high Tajima's *D* windows were selected. As we want to detect genomic regions with balancing selection acting during de-domestication, the regions have an average Tajima's *D*>1 or with high Tajima's *D* (>2.0) window in corresponding cultivated rice were further filtered.

### GO and metabolic pathway enrichment analysis

GO enrichment analysis was carried out using AgriGO^60^ with ‘*Oryza sativa* MSU6.1 non-TE' set as species background (http://bioinfo.cau.edu.cn/agriGO/). The *P*-value (hypergeometric) and false discovery rate (FDR) (Yekutieli) criteria of <0.0001 and<0.05, respectively, were used for the considered enrichment GO terms.

### Data availability

The short-read sequence data by this study have been deposited into the GenBank under the bioproject accession number PRJNA295802. The SRA accession numbers are SRR5337182-SRR5337186, SRR5337192-SRR5337424. All relevant data contained within the paper are available from the corresponding author on request.

## Additional information

**How to cite this article:** Qiu, J. *et al*. Genomic variation associated with local adaptation of weedy rice during de-domestication. *Nat. Commun.* **8,** 15323 doi: 10.1038/ncomms15323 (2017).

**Publisher's note:** Springer Nature remains neutral with regard to jurisdictional claims in published maps and institutional affiliations.

## Supplementary Material

Supplementary Information Supplementary Figures and Supplementary Tables

Supplementary Data 1 Genotyping and Tajima's *D* of *OsEXPA3* by Sanger method.

Supplementary Data 2 Demographic model parameters inferred for Chinese weedy rice.

Supplementary Data 3 Functionally characterized genes in divergent genomic regions (Z(F_ST_) ≥ 3) between weedy and cultivated rice.

Supplementary Data 4 Genomic windows with high Z-transformed F_ST_ values (≥ 3) of weedy rice versus cultivated rice populations.

Supplementary Data 5 GO enrichment terms for putative novel genes of weedy rice relative to cultivated rice.

Supplementary Data 6 Allele frequency differentiation (AFD) of standing and new SNPs for *japonica* and *indica* weedy rice, respectively.

Supplementary Data 7 Non-synonymous SNPs with high allele frequency difference (>0.7) between weedy rice and cultivated rice

Supplementary Data 8 Genes with high positive Tajima's *D* values in the Mb-scale regions of chromosome 5 of Jiangsu weedy rice.

## Figures and Tables

**Table 1:** Summary of phenotypes and genome sequencing data of Chinese weedy rice and local cultivated rice used in this study.

**Phenotype/sequencing**	**Weedy rice**	**Local rice**
Mean shattering level*	1.6	3.9
Awn (%)	43.9	2.6
Brown pericarp (%)	100	0
Dark seed hull (%)	42.6	19.7
Seed length width ratio	2.4	2.5
*japonica* type†	2.3	2.1
*indica* type†	2.5	3.3
Number of accessions sequenced‡	155	76
Total base-pairs generated (Gb)	1,074	541
Average genome coverage (× )	17.8	19.1
Mapped to reference genome (%)	92.5	95.4

^*^Based on shattering rate 1–5 at Fig. 1.

^†^Based on the phylogenetic tree (Fig. 2a).

^‡^Seven additional weedy rice from USA and South Korea were also sequenced.

**Table 2:** Characterization of the Mb-scale genomic region under balancing selection in weedy rice.

**Item\group type**	***indica*****(Jiangsu: 38 samples)**
Chromosome	Chr.5
Genomic position (Mb)	9.2–13.5
Region size (Mb)	4.4
Average π values (× 10^−3^)*	2.5/0.6
Top Tajima's values*	3.3/1.7
Average Tajima's values*	2.9/−1.8
Number of windows with Tajima's *D*>2*	43/0
Percentage of top 5% high Tajima's *D* windows	90.9%
Percentage of windows within *Z*(*F*st)≥3 across the genome	88.4%
Number of non-TE genes covered	213
Number of non-TE genes with Tajima's *D*>2*	132/4

Parameters were estimated in a 100 Kb scanning window along the whole reference genome.

^*^Weedy/all cultivated rice of *indica.*
