# Supplementary material for: Genomic variation associated with local adaptation of weedy rice during de-domestication
Source: Nat Commun. 2017 May 24;8:15323. doi: 10.1038/ncomms15323 (PMC5477509; doi:10.1038/ncomms15323)
Supplement: Supplementary Information — Supplementary Figures and Supplementary Tables [file ncomms15323-s1.pdf]

Supplementary Information

Supplementary Figures

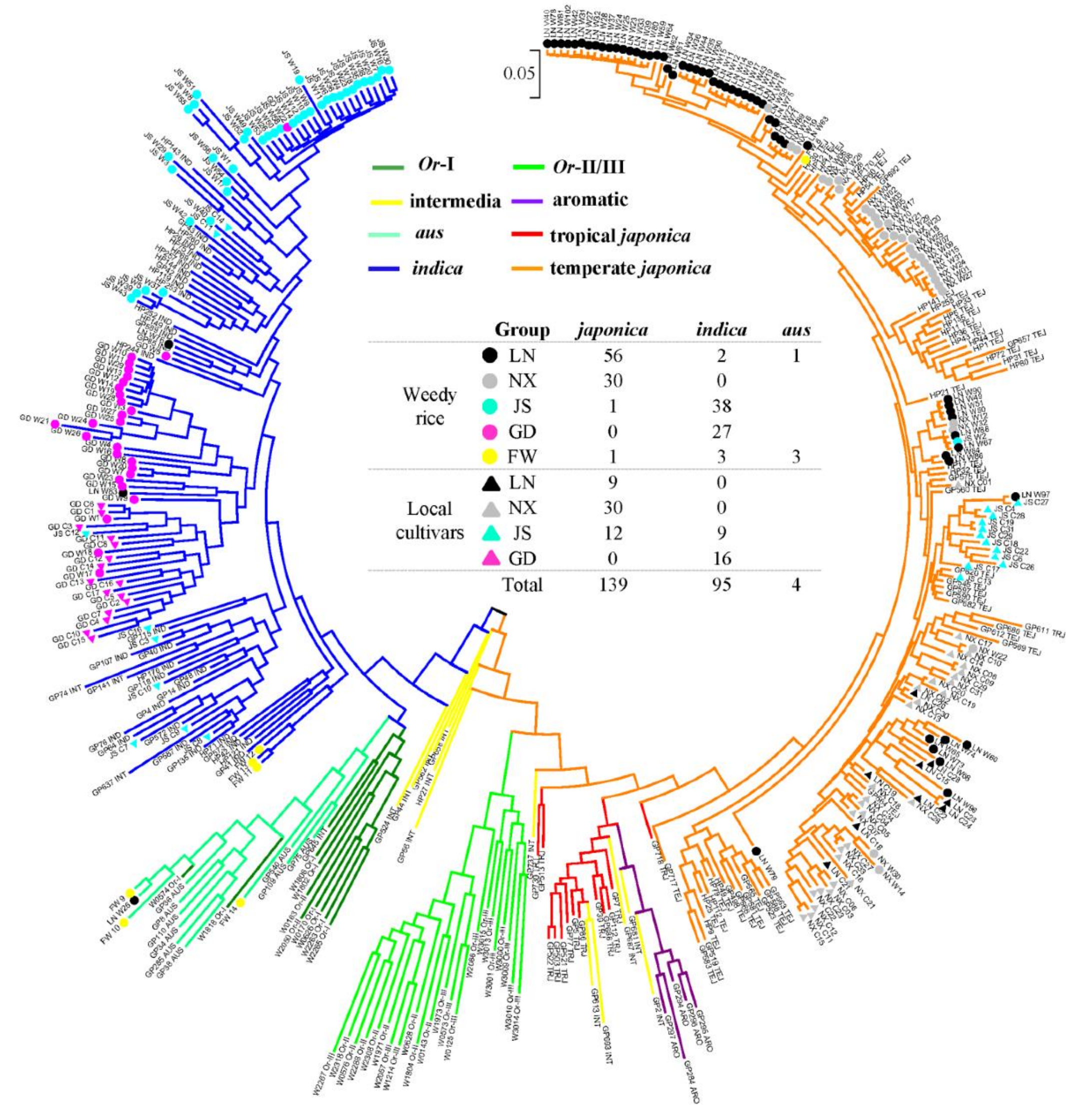

Supplementary Figure 1. Phylogenetic tree of weedy and cultivated rice with other *Oryza* species with detailed sample labels.

A maximum-likelihood phylogenetic tree of weedy and cultivated rice with other *Oryza* species. Different subgroups are coded with different colors: *O. rufipogon*: green for the *Or*-II and *Or*-III subgroups, dark green for the *Or*-I subgroup; *aus*: light green; *O. sativa* ssp. *japonica*: purple for aromatic *japonica* (ARO), red for tropical *japonica* (TRJ) and orange for temperate *japonica* (TEJ); *O. sativa* ssp. *indica* (IND): blue; intermediate type: yellow. The symbols for weedy and local cultivars from different geographical locations (LN for Liaoning, NX for Ningxia, JS for

Jiangsu, GD for Guangdong and FW for weedy rice sampled outside of China) are illustrated in the middle of the phylogenetic tree.

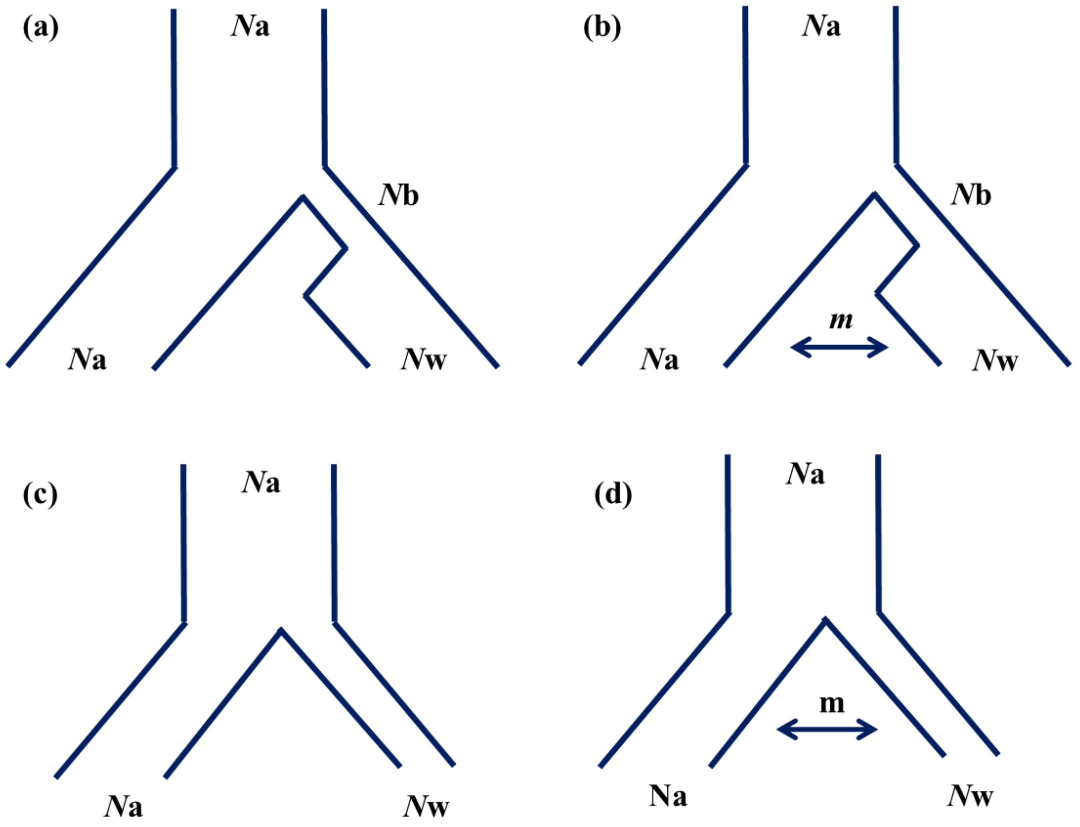

Supplementary Figure 2. Demographic simulation models considered for the origin of weedy rice.

Shown are the four possible demographic simulation models that were considered in this study: (a) weedy rice split from cultivar with bottleneck and does not allow migration with cultivar for the duration of the bottleneck; (b) weedy rice split from cultivar with bottleneck and can migrate with cultivar for the duration of the bottleneck; (c) weedy rice split from cultivar without a bottleneck and does not allow migration; (d) weedy rice split from cultivar without a bottleneck and allows migration.

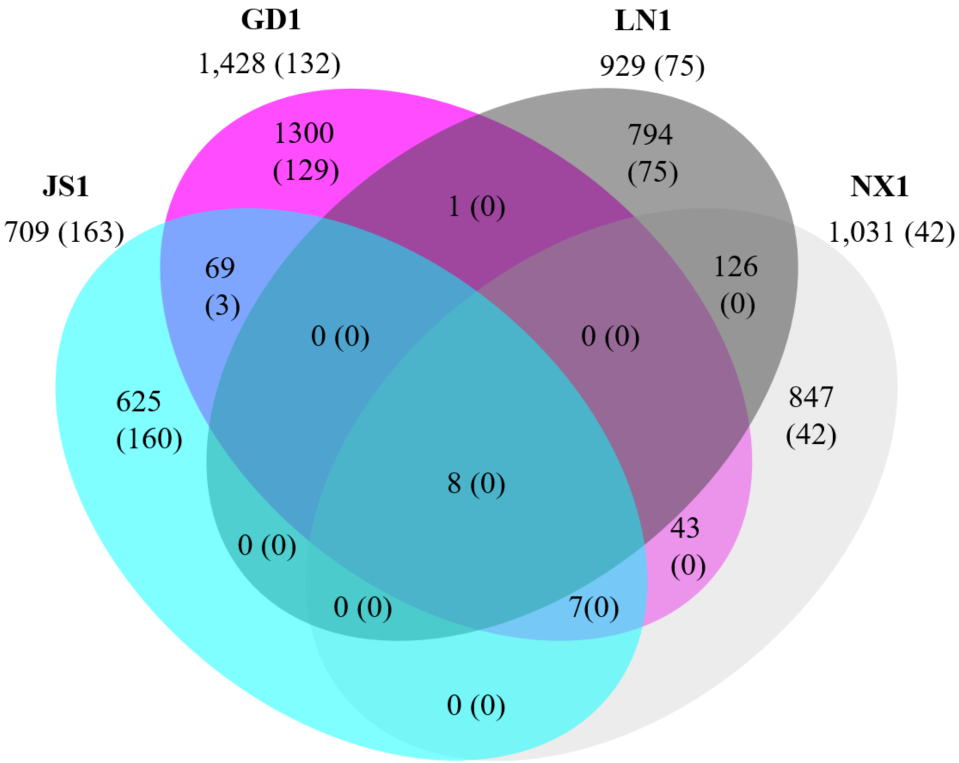

Supplementary Figure 3. Venn diagram showing the numbers of genes in the shared de-domestication signatures among the four weedy rice groups.

Venn diagram showing the numbers of genes in highly divergent windows ($Z(F_{ST}) \geq 3$) shared or unique among the four weedy rice groups. The different groups are color-coded as follows: *indica* weedy rice from Jiangsu (JS1) - blue; *indica* weedy rice from Guangdong (GD1) - purple; *japonica* weedy rice from Ningxia (NX1) - light grey; and *japonica* weedy rice from Liaoning (LN1) - dark grey. The number of SNPs considered is listed for each of the components and groups. The number of genes residing in domestication regions are presented in the parentheses for each component.

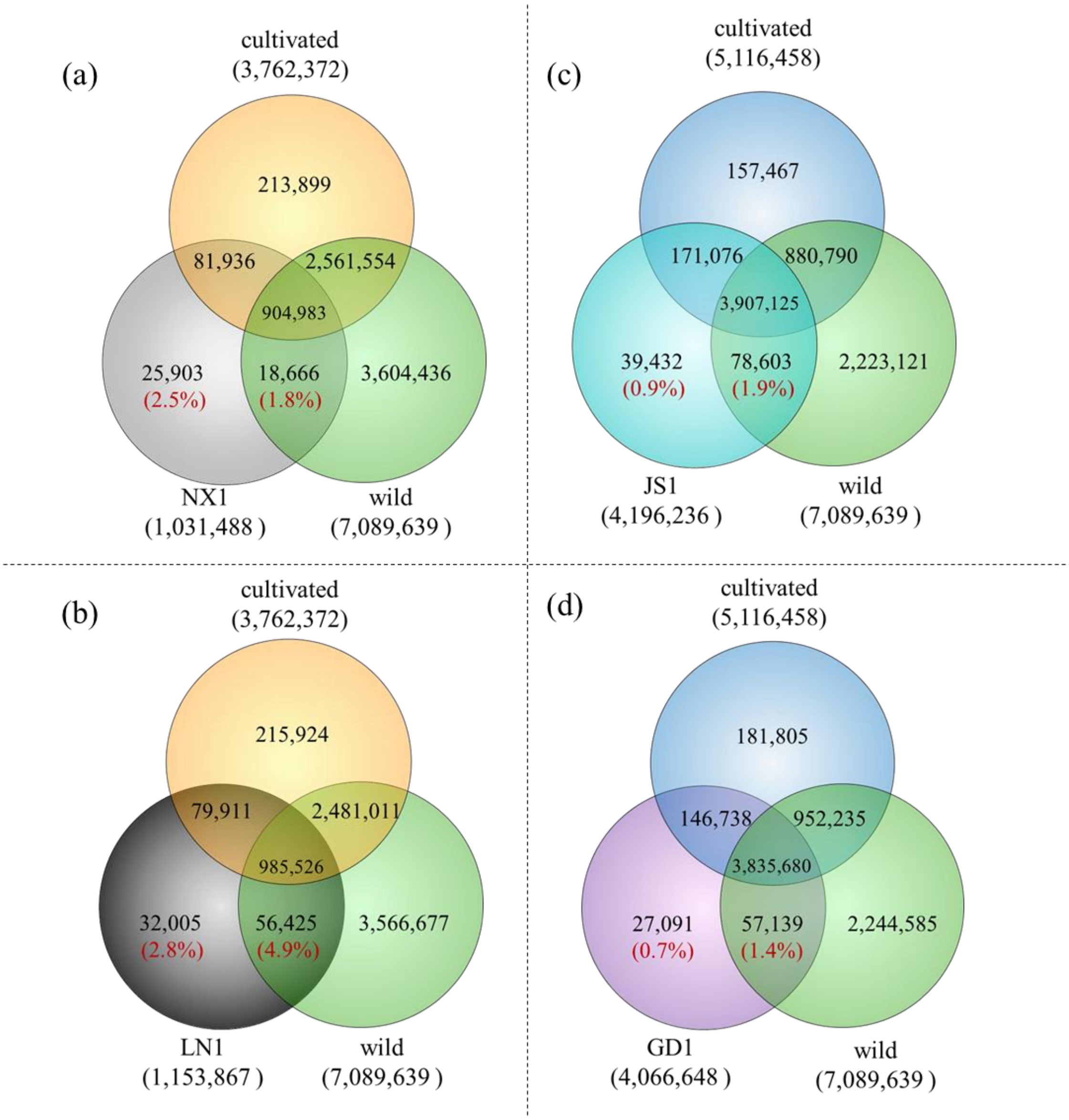

Supplementary Figure 4. Numbers of alleles across all SNPs characterized that are shared or unique among weedy, cultivated and wild rice. The different groups are color-coded as follows: blue and orange for *indica* and *japonica* cultivated rice, respectively; green for all wild rice (*Or*-I, *Or*-II, *Or*-III) used in this study. The weedy rice populations are color-coded as follows: NX1: grey (a), LN1: black (b), JS1: light blue (c), and GD1: purple (d). The number of SNPs is listed for each of the components and groups. Red font color indicates the percentages of unique SNPs in weedy rice compared to their cultivated rice counterpart.

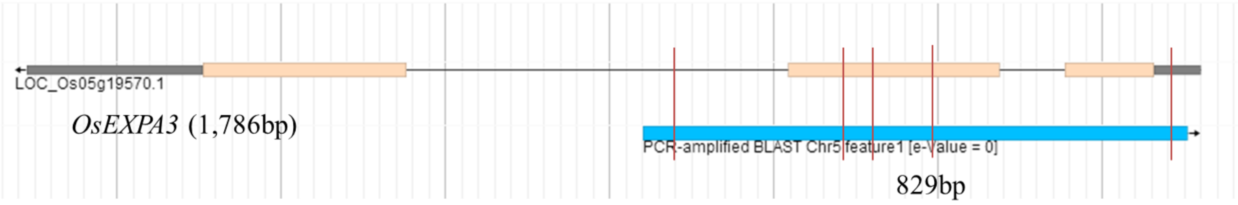

Supplementary Figure 5. Schematic representation of PCR-amplified sequence region within the *OsEXPA3* gene.

The PCR-amplified segment includes 5 SNPs based on next generation sequencing SNP calling. The red lines indicate the five SNPs within this region.

Supplementary Tables

Supplementary Table 1. Genotyping and Tajima's *D* of *OsEXPA3* by Sanger method.

(1) Primers designed for the two genes

Gene name	Gene ID	Related function	Marker name	Primer(5'-3')
OsEXPA3	LOC_Os05g19570	salinity tolerance	OsEXPA3-F	CCATCTCTGGTAGTCTGGTAGG
			OsEXPA3-R	AAATAGGGACGCTGTGATGCG

(2) Validation of genotyping

			Genotypes (with heterozygous SNPs)			Genotypes (without heterozygous SNPs)		
Gene	Number of samples	Number of SNPs	all	correct	accurancy	all	correct	accurancy
OsEXPA3	38	5	190	185	97.37%	185	185	100.00%

(3) Tests for selection for *OsEXPA3*

Group	Number of accessions	Sequence length (bp)	Number of seg. sites	θ_{π} (10^{-3})	θ_{w} (10^{-3})	Tajima's *D**
Jiangsu *indica* type weedy rice	38	829	5	2.48	1.44	1.87 (P = 0.029)

*Significance tested with 10,000 coalescent simulations.

Supplementary Table 2. Percent of windows with higher π or Tajima's *D* value in weedy than cultivated groups in weedy rice differentiated signature regions

Group	Number of accessions	Sequence length (bp)	Number of seg. sites	θ_{π} (10^{-3})	θ_{w} (10^{-3})	Tajima's *D**
Jiangsu *indica* type weedy rice	38	829	5	2.48	1.44	1.87 (P = 0.029)

Supplementary Table 3. Genomic regions (≥ 500Kb) under putative balancing selection in weedy rice but not cultivated populations.

Type	Genomic locations (Mb)		Size (Mb)	Average Tajima's *D* values	
	Chromosome	Window		Weedy rice	cultivars
JS1	chr5	3.5-4.0	0.60	3.19	0.12
JS1	chr5	9.2-13.5	4.40	2.89	-1.79
JS1	chr8	10.1-10.7	0.80	3.13	0.43
GD1	chr1	31.5-32.1	0.7	2.93	0.06
GD1	chr8	11.1-12.0	1	3.3	-0.8
GD1	chr8	12.2-12.6	0.5	3.07	-0.4
GD1	chr8	14.1-14.5	0.5	3.25	-0.89
NX1	chr4	0-0.8	0.9	2.51	0.27
NX1	chr4	11.4-11.9	0.6	3.38	-1.03
NX1	chr7	8.2-8.8	0.7	2.85	-1.29
NX1	chr7	9.2-9.6	0.5	2.94	-1.06
NX1	chr9	10.9-11.3	0.5	2.92	0.4
LN1	chr1	31.4-31.8	0.5	3.84	-0.15
LN1	chr2	2.7-3.3	0.7	3.78	0.02
LN1	chr3	8.8-9.2	0.5	3.6	-0.29
LN1	chr3	35.2-35.7	0.6	3.67	-1.56
LN1	chr5	4.8-5.3	0.6	3.25	-0.94
LN1	chr8	7.5-8.2	0.8	3.4	-0.48
